# Assessing the influence of perfusion on cardiac microtissue maturation: A heart‐on‐chip platform embedding peristaltic pump capabilities

**DOI:** 10.1002/bit.27836

**Published:** 2021-06-01

**Authors:** Daniela Cruz‐Moreira, Roberta Visone, Francisco Vasques‐Nóvoa, António S. Barros, Adelino Leite‐Moreira, Alberto Redaelli, Matteo Moretti, Marco Rasponi

**Affiliations:** ^1^ Department of Electronics, Information and Bioengineering Politecnico di Milano Milano Italy; ^2^ Cardiovascular Research and Development Center, Faculty of Medicine University of Porto Porto Portugal; ^3^ Department of Surgery and Physiology, Faculty of Medicine University of Porto Porto Portugal; ^4^ Instituto de Investigação e Inovação em Saúde (i3S) University of Porto Porto Portugal; ^5^ Cell and Tissue Engineering Laboratory IRCCS Istituto Ortopedico Galeazzi Milan Italy; ^6^ Regenerative Medicine Technologies Lab Ente Ospedaliero Cantonale (EOC) Lugano Switzerland; ^7^ Present address: Daniela Cruz‐Moreira, 3B's Research Group–Biomaterials, Biodegradables and Biomimetics University of Minho, AvePark‐Parque de Ciências e Tecnologia, Zona Industrial da Gandra Barco Portugal

**Keywords:** heart, heart‐on‐chip, maturation, mechanotransduction, perfusion

## Abstract

Heart‐on‐chip is an unprecedented technology for recapitulating key biochemical and biophysical cues in cardiac pathophysiology. Several designs have been proposed to improve its ability to mimic the native tissue and establish it as a reliable research platform. However, despite mimicking one of most vascularized organs, reliable strategies to deliver oxygen and substrates to densely packed constructs of metabolically demanding cells remain unsettled. Herein, we describe a new heart‐on‐chip platform with precise fluid control, integrating an on‐chip peristaltic pump, allowing automated and fine control over flow on channels flanking a 3D cardiac culture. The application of distinct flow rates impacted on temporal dynamics of microtissue structural and transcriptional maturation, improving functional performance. Moreover, a widespread transcriptional response was observed, suggesting flow‐mediated activation of critical pathways of cardiomyocyte structural and functional maturation and inhibition of cardiomyocyte hypoxic injury. In conclusion, the present design represents an important advance in bringing engineered cardiac microtissues closer to the native heart, overcoming traditional bulky off‐chip fluid handling systems, improving microtissue performance, and matching oxygen and energy substrate requirements of metabolically active constructs, avoiding cellular hypoxia. Distinct flow patterns differently impact on microtissue performance and gene expression program.

## Graphical abstract

A heart‐on‐chip platform integrating an automated peristaltic pumping system is developed as an accurate tool to study the influence of perfusion on cardiac tissue maturation. The study focus on the impact of flow rate on the temporal dynamics of cell morphology, cellular organization, contractile behavior, together with a comprehensive analysis of relevant transcriptional profile.

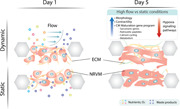

## 1 INTRODUCTION

Miniaturized in vitro models offer unprecedent opportunities to unravel the rules that govern biological organization (Visone et al., 2016). Specifically, heart‐on‐chip technology has gained attention overtime due to its potential to guide tissue development by providing biochemical and biophysical signals tailored to recapitulate the complex native myocardial tissue faithfully (Marsano et al., 2016; Mathur et al., 2015; Visone et al., 2018).

Advances of heart‐on‐chip technology have been pursued by introducing key physiological stimuli, namely, mechanical (Marsano et al., 2016) and electrical (Visone et al., 2018), positively impacting microtissue maturation and performance.

The myocardium is the most densely vascularized tissue in the human body and operates near the maximal oxygen extraction at rest (Laughlin & Tomanek, 1987). Due to its large microvascular exchange surface area, the myocardium is faced with a very high interstitial fluid filtration rate, at least an order of magnitude greater than most other organs (Uwe et al., 2001). Therefore, in vitro cardiac models, often represented by high‐density three‐dimensional (3D) constructs of metabolically demanding cells, must ensure adequate delivery of oxygen and energy substrates to avoid cellular stress and enhance system reproducibility.

Indeed, impaired diffusional transport in high cellular density systems has been associated with gradients in oxygen concentration, cell density, viability, and function (Radisic et al. 2004, 2006; Richards et al., 2020). To uniformly regulate the process of cell feeding through the construct, perfusion systems have been integrated into cell culture platforms (Jackman et al., 2016; Radisic et al., 2004; Visone et al., 2018). However, regarding microscale systems, the effect of perfusion on maturation and contractile performance was not adequately evaluated due to the lack of appropriate controls (Kobuszewska et al., 2017; Mathur et al., 2015). Another major limitation of these systems was the use of external perfusion tools that compromise the throughput and reproducibility of the studies since they limit the number of parallel experiments. Therefore, a comprehensive study highlighting the role of perfusion on cardiac microtissue structural, functional, and transcriptional maturation is still missing.

We hereby describe a new microfluidic platform to investigate the influence of controlled perfusion conditions on developmental and functional maturation of 3D cardiac ventricular microtissues. The device design integrates an on‐chip peristaltic pumping system, allowing an automated, fine control over flow profiles on the side channels that flank a central 3D cardiac culture chamber. The system was designed to be completely automated and compatible with standard cell culture conditions (i.e., controlled atmosphere of humidified CO_2_ incubator) while accurately controlling cell culture medium perfusion.

## 2 MATERIALS AND METHODS

### 2.1 Design of a microfluidic platform for perfusion of 3D cardiac microtissues

An on‐chip pump was designed and built to perfuse cell culture medium to feed 3D cardiac microtissues. The upper cell culture layer has an overall footprint of 2 mm by 3 mm and a height of 100 µm. In the central part, two arrays of hexagonal micropillars define three compartments: the central region representing the cell culture compartment (300 µm wide) is enclosed by the arrays of posts, flanked by two side perfusion compartments. The gap between micropillars is 50 µm to allow cell‐laden gel confinement during injection (Huang et al., 2009; Marsano et al., 2016; Ugolini et al., 2017; Visone et al., 2018). The bottom control layer contains three blind channels (100 µm‐wide) intended to act as independent control lines. Control and fluidic layers are separated by a 34–45 µm thick polydimethylsiloxane (PDMS) membrane responsible for deflecting upon increase of pressure and closing the fluidic channel (Gómez‐Sjöberg et al., 2007; Kellogg et al., 2014; Unger et al., 2000).

### 2.2 Microfabrication

The device layout was drawn using computer‐assisted design (CAD) software (AutoCAD, Autodesk Inc.). The corresponding optical masks featuring valves, chamber, and control layer for photolithography were printed at high resolution (64,000 DPI) on a polyester film (JD Photodata) used as photomask.

The microfluidic platform was conceived as a modular system, consisting of a cell culture layer and a pressure‐actuated control layer. The mold for each layer was produced on silicon wafers by photolithography techniques. The photoresist was spin‐coated, postbaked, and developed according to suppliers' instructions. PDMS‐based devices were produced by multilayer soft lithography. The thickness of the membrane between the two layers was defined by spin coating uncured PDMS on the control mold. Both control and cell culture layers were precured, aligned and baked at 80°C to achieve irreversible bonding. Details of the chip fabrication procedure can be found in Supplementary Information.

### 2.3 Development of a custom‐made control system

The automation system is composed of a series of miniature solenoid valves (MH1, FESTO) connected directly to the control channels of the chip and a pressure control block constituted by a pressure source and a pressure regulator. An electronic control block formed by a microcontroller and an amplification board was used to actuate the valves. Custom‐made software was also developed to operate the chip by an intuitive touchscreen‐based graphical user interface (GUI).

The multiple valve manifold is controlled as two sets of three valves each, allowing the operation of two different sets of chips running at different flow rates in parallel, using the same control system. Each solenoid valve can switch between its usually close state at atmospheric pressure (on‐chip valve open) and actively open (on‐chip valve closed) that let the pressure rise to 20 psi.

### 2.4 Evaluation of flow output

The on‐chip pump performance was investigated by correlating the driven frequency of the pump with the flow rate generated at constant back pressure (Lai & Folch, 2011). The volumetric flow rate was calculated as a cylindrical volume as a function of the distance traveled by the fluid front per time. These values were analyzed using scientific graphing and statistic software Prism (GraphPad Software Inc.). Detailed method to determine flow output can be found in Supplementary Information.

### 2.5 Ethics statement

In this study, cells were isolated from hearts of 2 day‐old neonatal Sprague–Dawley rats (Charles River) collected from animals involved and euthanized in another study unrelated to the ongoing research. All the applicable international, national, and/or institutional guidelines for using these animals were followed. The Institutional Animal Care and Use Committee of the San Raffaele Scientific Institute (IACUC 795) approved the study designed for this group of animals. All procedures on animals involved in this study were following the ethical standards.

### 2.6 Cell isolation

Neonatal rat ventricular myocytes (NRVM) were isolated from 2‐day‐old Sprague–Dawley rats following the previously described protocol (Sadeghi et al., 2017). Briefly, after heart dissection, atria were discarded while ventricles were quartered and digested overnight in 0.06% (w/v) trypsin (Sigma‐Aldrich) in Hank's balanced salt solution (HBSS, Gibco) to lose the tissue. Trypsin was neutralized by transferring the heart pieces to Dulbecco's modified essential medium‐high glucose (DMEM, 4.5 g/L glucose) supplemented with 10% v/v fetal bovine serum (FBS, Hyclone), 100 U/ml penicillin, 100 μg/ml streptomycin and 10 mM 4‐(2‐hydroxyethyl)‐1‐piperazineethanesulfonic acid (HEPES, Gibco) and swirled for 5 min at 37°C. Ventricular heart pieces were then transferred in 0.1% (w/v) collagenase type II (305 u/mg) (Worthington Biochemical Corporation) in HBSS and two rounds of digestions were performed at 37°C at 80 rpm for 10 min each, followed by mechanical disruption of the tissue. Cells were pelleted at 200*g* for 5 min and resuspended in supplemented DMEM. To enrich the population of cardiomyocytes, cells were preplated for 1 h and subsequently, the nonadherent cells were transferred to a second culture flask and plated overnight in DMEM (supplemented with 10% v/v FBS [Hyclone], 100 U/ml penicillin, 100 μg/ml streptomycin, 10 mM HEPES, 2 µg/ml insulin [Sigma], 50 µg/ml ascorbate [Sigma] and 5 µM Cytosine‐B‐d‐arabino‐furanoside hydrochloride [AraC, Sigma]).

### 2.7 Cell seeding and culture

The isolated cardiac population was embedded in a fibrin gel at a density of 1 × 108 cells/ml and injected into the microfluidic platform to cast fibrin gel constructs. In detail, cells were suspended in DMEM containing fibrinogen in 20 mM HEPES buffered saline to achieve a final fibrin gel solution of 4 mg/ml fibrinogen. A final concentration of 0.4 U/ml of thrombin allowed the crosslinking of fibrin gel. Cell‐laden fibrin gel was carefully mixed using a pipette, and while in its liquid‐phase, it was injected in the cell culture channel of the fluidic chamber. The gel was incubated for 20 min (37°C; 5% CO_2_) to allow complete gel polymerization. Media reservoir was attached to the inlet of the microfluidic chip. It was then pressurized at 20 psi with 5% CO_2_ air source to feed the construct and prime the channel with DMEM (supplemented with 10% v/v FBS, 100 U/ml penicillin, 100 μg/ml streptomycin, 10 mM HEPES, 2 µg/ml insulin, 50 µg/ml ascorbate, and 6 mg/ml of aminocaproic acid [Sigma]) defined as supplemented growth media. Cardiac microtissues were cultured for 5 days, being perfused for 30 s each 10 min under low flow (LF; 31 µl/min) and high flow (HF; 48 µl/min) rate. At the interface, the average wall shear stress was estimated to be 1.4 and 2.16 dyn/cm^2^ for LF and HF, respectively. After 30 s of perfusion, cell culture medium channels were completely filled with fresh medium in both conditions. Static cultures were used as control.

### 2.8 Functional analysis of cardiac microtissues

Cardiac microtissues were optically monitored daily after seeding, on an inverted optical microscope (Olympus IX‐71) equipped with an environmental chamber for live‐cell imaging.

After 1 and 5 days in culture, cardiac microtissues were paced using stainless steel electrodes connected to a previously described custom made electrical stimulator (Visone et al., 2018). Pacing signal was a monophasic square pulse of 4 ms duration with a tunable frequency (1–10 Hz) and amplitude (0–10 V, 0.1 V resolution). Excitation threshold (ET) was measured by adjusting the signal in voltage and frequency, respectively (Tandon et al., 2010). ET is the minimum amplitude that induces sustained synchronous contractions of the microtissue at 1 Hz frequency.

Mean contraction velocity of cardiac microtissues paced at 1 Hz was assessed. Bright‐field microscopy videos of cardiac microtissues were analyzed using a custom‐made software developed at Boston University (http://gladstone.ucsf.edu/46749d811) (Huebsch et al., 2015). Briefly, a macroblock tracking strategy was used to create motion vector fields generated by block matching. The movement of a given pixel macroblock at a specific frame is computed by matching a block of pixels to an identically sized block of pixels in the next frame. The generation of motion vectors allows the calculation of motion velocity, characterized by the difference in intensity and time of subsequent contraction and relaxation peaks.

### 2.9 Immunofluorescence staining

Immunofluorescence analysis was performed at Day 0 and after 5 days of culture on perfused and static cardiac microtissues directly within the microdevices. Briefly, samples were fixed in 4% (v/v) paraformaldehyde (PFA, Santa Cruz) for 15 min and incubated with a solution of 0.2% (v/v) Triton X‐100, 2% (w/v) bovine serum albumin (BSA) and 1% (v/v) FBS in phosphate buffer saline (PBS) for 1 h at room temperature.

Samples were incubated at 4°C overnight with primary antibody, Troponin I (Santa Cruz). Subsequently, samples were incubated in the dark at 4°C for 6 h with fluorescein isothiocyanate (FITC)‐conjugated secondary antibody. 4′,6‐diamidino‐2‐phenylindole (DAPI) counterstaining was used to identify cell nuclei by incubating the immunostained samples for 10 min at room temperature.

### 2.10 RNA extraction and real‐time polymerase chain reaction (PCR)

The extraction of total mRNA from cardiac microtissues was made by selective binding to membranes of silica gel (RNeasy Mini Kit, Qiagen) according to the manufacturer's instructions. Briefly, 100 ng of total RNA were submitted to reverse transcription (SuperScript IV Reverse Transcriptase, Thermo Scientific Fisher) according to the manufacturer's instructions. Ten percent of the RT product was subjected to real‐time PCR (StepOnePlus™ Real‐Time PCR System, Thermo Scientific Fisher) using SYBR green as marker (PerfeCTa SYBR Green SuperMix, Quantabio). A standard curve was generated from the correlation between serial dilutions of a random sample and PCR cycle threshold (obtained by method of the maximum second derivative). The results were analyzed after normalization for internal control 18 S ribosomal RNA (18 S) and expressed in multiples of an arbitrary unit, which corresponds to the average control group (Ctrl Day 1). Analyzed genes and corresponding primers are listed in Table S1.

### 2.11 Statistical analysis

All data were statistically analysed through GraphPad Prism software. Data were represented and expressed with mean ± SEM. Statistical analysis was performed using one‐way and two‐way analysis of variance for multiple group comparisons followed by Holm–Sidak's method for post hoc comparisons.

Hierarchical heatmap clustering was applied to uncover characteristic gene patterns as a function of the experiment. The different genes are grouped according to the Euclidean distance using complete linkage (farthest neighbors) using the pheatmap package in the R environment (Kolde, 2019).

## 3 RESULTS

### 3.1 Design and characterization of the microfluidic platform for perfusion of 3D cardiac microtissues

**Figure 1:**
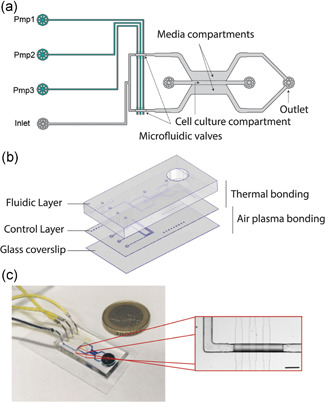
(a) Cells, medium, and reagents are contained in the upper cell culture layer (gray), whereas elastic membrane valves are created by the bottom pressure‐actuated control lines green); (b) exploded view and schematic of the microfluidic device configuration. The microfluidic device integrated with on‐chip peristaltic pump consists of a fluidic layer for the 3D cell culture, a control layer that controls the fluid perfusion and a glass cover slide that seals the control layer. The thin and thick layers are bonded through thermal bonding overnight at 80°C, while the coverslip is bonded to the device through air plasma; (c) picture of a representative on‐chip pump. Coin‐size microfluidic device connected to the medium reservoir (represented in blue) and the actuation lines (represented in yellow). Different color dies were used to demonstrate the layer separation through a thin membrane. Magnification of peristaltic pump region. All valves are open. Scale bar: 200 µm [Color figure can be viewed at wileyonlinelibrary.com]

The heart‐on‐chip platform embedding a peristaltic pump for perfusion of 3D cardiac microtissues was conceived to be fully compatible with standard cell culture facilities. The on‐chip pump was designed as a modular system (Figure 1a), consisting of a cell culture layer (gray) coupled with a pressure‐actuated control layer (green) placed underneath. In the cell culture layer, two arrays of hexagonal posts divide the cell culture channel from the medium channels. This caging structure avoids extravasation of the cell‐laden prepolymer solution during the injection and crosslink of the hydrogel (Marsano et al., 2016; Visone et al., 2018; Wu et al., 2008). A media reservoir is connected to the chip in a binary tree manifold that guarantees equal hydraulic resistance in all branches. A dedicated output well at the end of the cell culture chamber is used for waste collection. The control layer contains three dead‐end channels that act as control lines. Microfluidic channels in these two layers are separated by a thin deformable membrane able to open and close channels by regulating the pressure in the control lines (Gómez‐Sjöberg et al., 2007; Unger et al., 2000). In particular, the intersection of the three control lines with the fluidic layers form two parallel on‐chip peristaltic pumps, each composed of three valves in series located at the root of the cell culture chamber. Peristaltic pumps allow the metering of precise doses of culture media equally into both media compartments. The platform was successfully fabricated through photolithography and multilayer soft lithography techniques in polydimethylsiloxane (PDMS) (Figure 1b). The fluidic layer has mostly a rectangular cross‐section of 101.2 ± 3.6 µm height, except on the regions of valves that acquired a round cross‐section through reflow of positive photoresist (maximum height of the rounded channel is 42.8 ± 2.9 µm). Channels in the control layer have a rectangular cross‐section (21.1 ± 0.6 µm high).

The two PDMS layers were bonded through thermal bonding of PDMS, thus, allowing the proper alignment of the fluidic and control channels. Figure 1c shows a representative image of the device, connected to the control system through Tygon tubing, together with an inlet where the control lines are aligned to the fluid channel of the flow layer.

**Figure 2:**
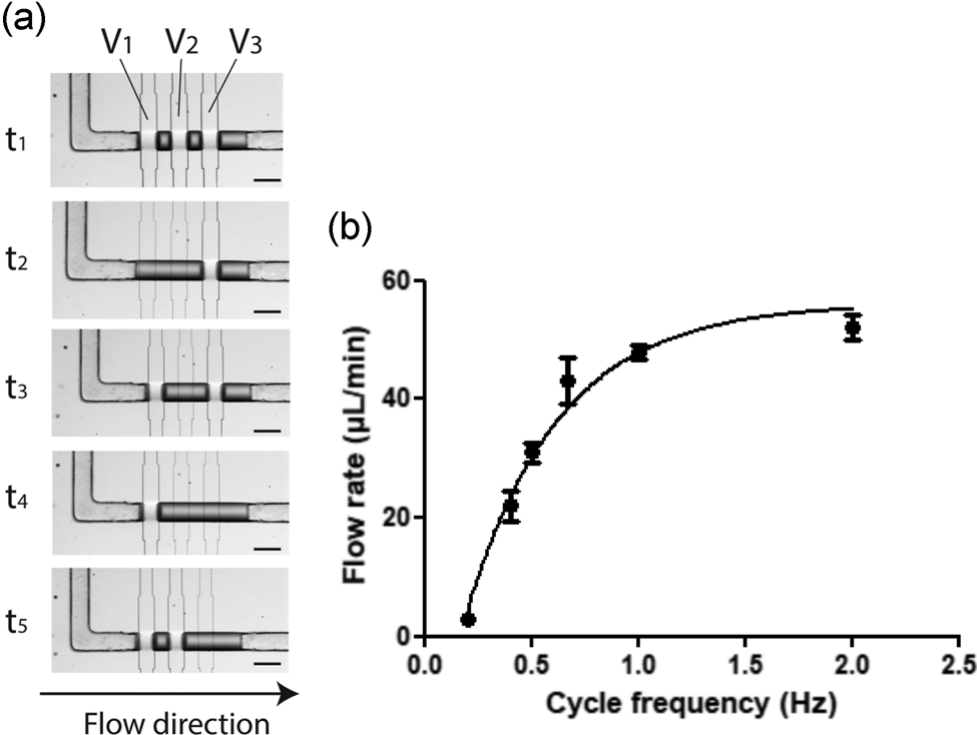
(a) Operation of 5‐step cycle within on‐chip pump. Microphotographies of the valve actuation pattern of the peristaltic pump. At rest, the three valves are close to prevent leakage. When pumping, the fluid is allowed to enter the valve region and is sequentially pushed in the output direction. At least one valve is always close during the pumping sequence to prevent backflow. Scale bar: 200 µm. (b) Flow rate curve. The on‐chip pump's performance was investigated by correlating the driven frequency with the flow rate generated at a fixed back pressure

The on‐chip peristaltic pump was actuated by sequencing the three microfluidic valves in a 5‐step cycle, enabling the fluid motion within the channel (Figure 2). The delay between each of the five consecutive steps dictates the pumping frequency and, consequently, the flow rate imposed in the chip. To characterize the on‐chip valves performance integrated with the control system, the pump was actuated and imaged through optical microscopy. As observed in Figure 2a, when a microvalve is actuated, the underlying control channel inflates the planar membrane to form a hemispherical bulge that completely closes the semi‐circular flow channel above. Each control line was actuated independently by controlling a 3‐way solenoid valve (MH1 valves, Festo Group).

The on‐chip peristaltic pump performance was assessed by measuring the volumetric flow rate corresponding to the driven frequency of a complete cycle of the pump. As illustrated in Figure 2b, the flow rate increases with valve actuation frequency in a nonlinear fashion. Indeed, the curve, analysed using nonlinear regression, could approximate a logarithmic tendency (*R* ^2^ = 0.98) representing a saturation area at high frequencies due to valve response time limit (Au et al., 2011; Wu et al., 2008).

Additionally, this heart‐on‐chip was designed respecting specific dimensions and following the guidelines to easily integrate other features developed by our group, adding in this manner complexity to the stimulation system. Specifically, this chip fits the bottom chamber described by Marsano et al., and allows the user to introduce uniaxial mechanical stimulation on demand (Marsano et al., 2016). Moreover, guiding channels can be easily added to the chip geometry to position microelectrodes and provide either electrical stimulation (Visone et al., 2018) or online continuous electrical recording (Visone et al., 2021).

### 3.2 Cell seeding and development of tissue architecture

The initial cell population used to generate the cardiac microtissues was composed mainly by cardiomyocytes (71 ± 9%), identified as positive Troponin I, while nonmyocytes constituted 29 ± 9% of the population (Figure S1). Primary NRVM were embedded in fibrin gel at a concentration of 1 × 105 cells/µl, seeded in 3D conformation within the microfluidic platform, and cultured for 5 days under different profiles of medium perfusion: 31 µl/min (corresponding to 0.5 Hz frequency of a complete cycle of the pump, or LF rate), 48 µl/min (corresponding to 1 Hz frequency of a complete cycle of the pump, or HF rate). Static cultures were used as control (Ctrl).

**Figure 3:**
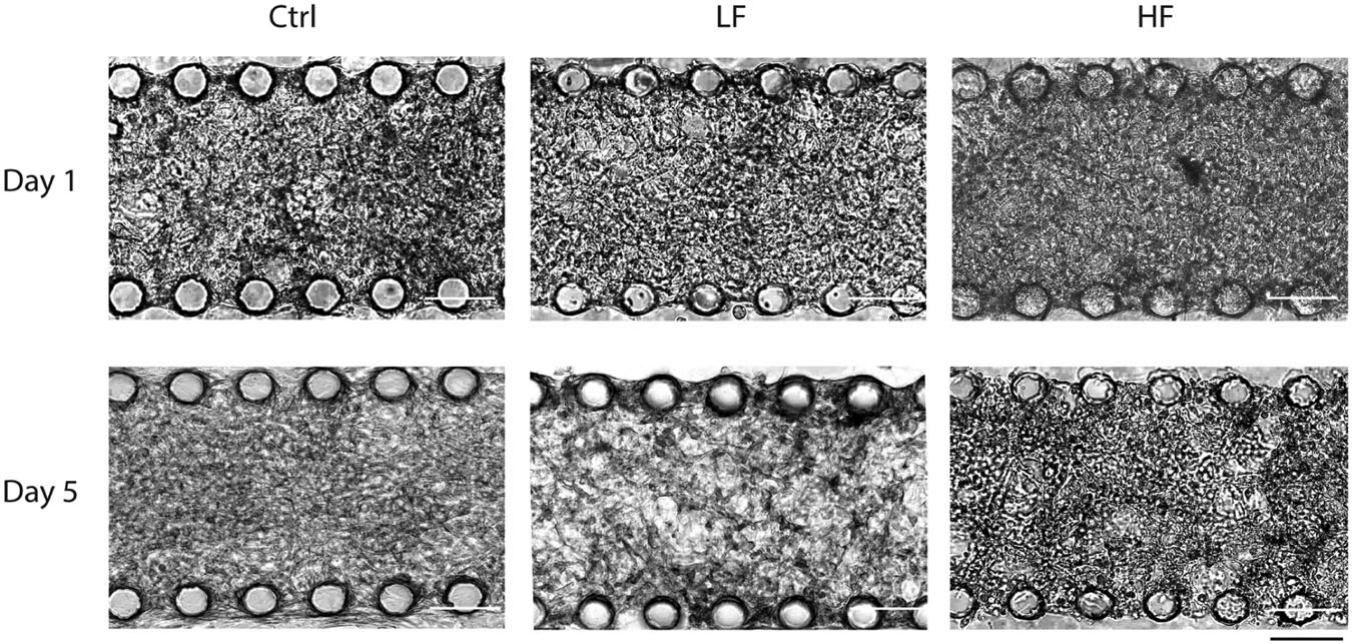
Representative bright‐field microphotographs of cardiac microtissues cultured within the on‐chip pump platform. NRVM embedded in fibrin hydrogel were cultured for 5 days and were perfused at different flow rates. Initially, microtissues present a compact configuration of rounded cells. ECM remodeling, cell elongation, and connection are evident throughout the experiment. Scale bar: 100 µm. NRVM, neonatal rat ventricular myocytes

Due to high cell density, the cultured cardiac tissue looked compact, with no visible spaces among the cells. After cell seeding, extracellular matrix (ECM) remodeling and tissue compactness were visually assessed, and corresponding bright‐field images were acquired (Figure 3). The engineered cardiac tissue was still compact on the day following seeding, and cells were uniformly distributed among all the groups, with some cell spreading being observed (Figure 3).

After 5 days in culture, cells exhibited an elongated morphology, and the ECM was visibly remodeled, with evident differences among the conditions (Figure 3). In particular, cardiac microtissues cultured in static conditions presented a slightly more compact structure, suggesting a minor remodeling of the ECM. However, in the perfused cardiac microtissues, the remodeling was evident with more pronounced effects in HF microtissues.

**Figure 4:**
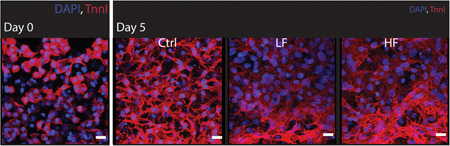
Representative immunofluorescence microphotographs of cardiac microtissues cultured within on‐chip pump platform. Cardiac microtissues were stained for Troponin I and DAPI at Day 0 and after 5 days in culture. On Day 0, cardiomyocytes present a round configuration. After 5 days in culture, perfused cardiomyocytes express a higher activity regarding ECM remodeling and the cell size is considerably higher than static conditions. Scale bar: 20 µm [Color figure can be viewed at wileyonlinelibrary.com]

Cardiomyocyte maturation was observed in all conditions, as evidenced by the elongated morphology compared with cell morphology at Day 0, represented by specific immunostaining for cardiac troponin I (Figure 4). On Day 5, the irregular shape and random arrangement of NRVM were similar in all conditions. Interestingly, a slightly higher concentration of NRVM in the proximity of the post rows that limit the central culture channel was also observed (Figure S2) (Marsano et al., 2016; Visone et al., 2018, 2021). Moreover, in the perfused conditions, both cardiomyocyte size and the area of cell–cell connections were evidently increased (Figure 4). NRVM exposed to perfusion showed a more complex organization of the sarcomeric structures at the ultracellular level, as highlighted by the cytoskeletal striation of the Troponin I staining. Although microtissues cultured under static conditions appear to overall preserve the original fibrin gel, after 5 days, an evident remodeling of the embedding hydrogel was observed in the perfused microtissues, especially on the HF conditions.

### 3.3 Assessment of contractile behavior

**Figure 5:**
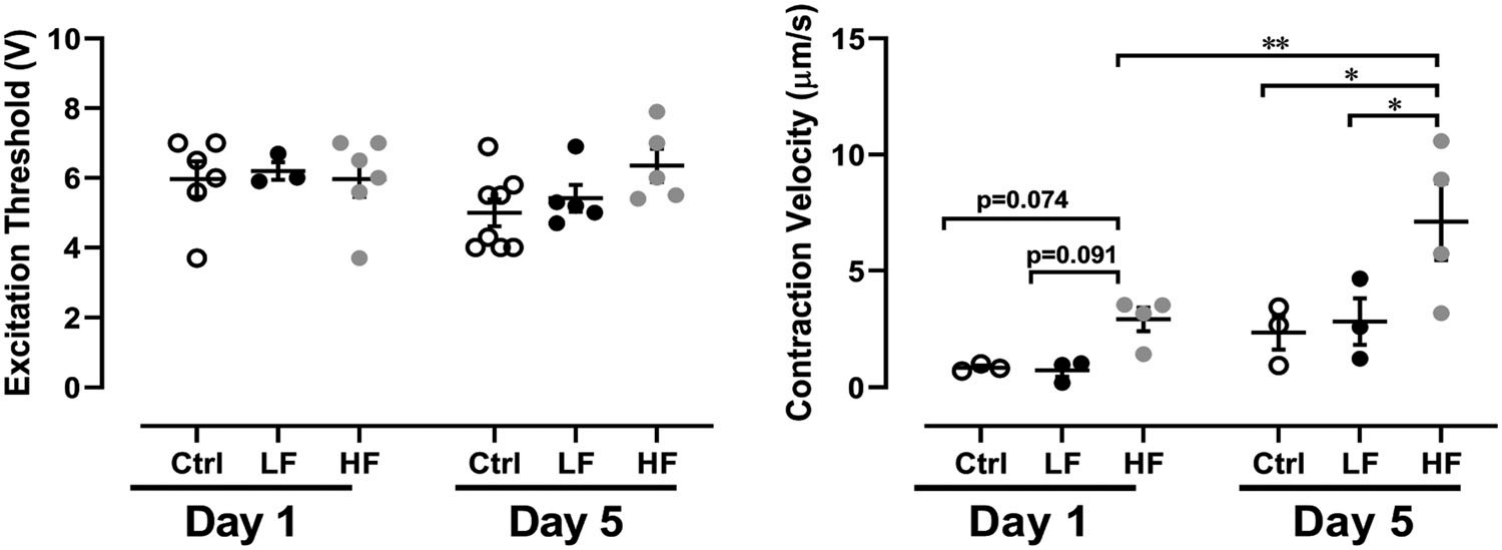
Pacing tests performed to assess ET parameters, evaluating the influence of medium perfusion in the electrical excitability of cardiac microtissues. Video analysis were performed to assess the contraction velocity, to evaluate the influence of medium perfusion in beating functionality of cardiac microtissues (*n* > 4 biologically independent samples. Statistically significance was determined by Bonferroni's multiple comparison test, *** *p* <** **0.001).

Pacing tests were performed to assess the influence of medium perfusion on the electrophysiology of the cardiac microtissues (Figure 5). In response to electrical stimulation, cardiac constructs from all groups were induced to contract synchronously. There was no significant difference in the ET measured for the three groups, both at Day 1 and Day 5, even though a slight decrease in ET was observed with time in culture in all groups. The effect of perfusion on contractility was assessed by analyzing the beating profile, specifically beating velocity of paced constructs (Figure 5). Microtissue contraction velocity during pacing at Day 5 revealed an enhanced contractile performance in the HF group when compared with both LF and control. Furthermore, the contraction velocity of HF microtissues was significantly higher after 5 days in culture.

### 3.4 Influence of flow rate on gene expression of maturation markers

**Figure 6:**
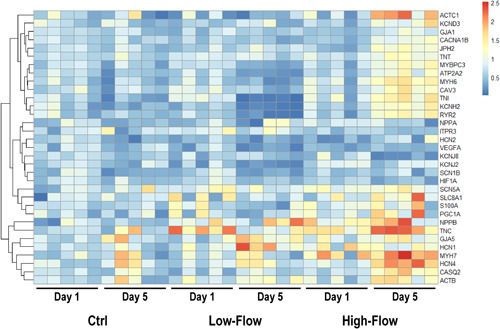
Heatmap of differential gene expression. Heatmap showing the integrated expression change in all groups. Rows represent genes and columns represent individual samples. *N* = 5 biologically independent samples/group [Color figure can be viewed at wileyonlinelibrary.com]

A comprehensive gene expression panel evaluation, including genes related to intercellular connections, pacemaker channels, sodium and potassium channels, calcium cycling, sarcomere structure, cell membrane and cytoskeleton, response to hypoxia and metabolism, was performed to assess microtissue maturation and transcriptional response to culture conditions (Figure 6). On Day 1, despite the absence of widespread alterations in gene expression, perfused microtissues showed an increased expression of Troponin C (TNNC1; Figure S3) and a lower expression of hyperpolarization‐activated cyclic nucleotide–gated (HCN) channel 2 (HCN2; Figure S4) and VEGF A (VEGFA), when compared with static conditions (Figure S5). In comparison with Day 1, gene expression in static conditions (Ctrl) at Day 5 showed only minor downregulation of sodium (SCN1B) and potassium (KCNH2) channels (Figure S6). In perfused microtissues, however, gene expression profile was significantly altered at Day 5 when compared with Day 1 (Figure 6). Importantly, the direction of gene expression changes with time did not overlap entirely between LF and HF perfused microtissues. While there were concordant changes between LF and HF groups in response to perfusion in hypoxia‐driven gene expression (HIF1A; Figure S5), discordant changes could be observed in genes related to sarcomere structure (MYBPC3, TNNC1, and TNNI3; Figure S3) and calcium cycling (ATP2A2 and RYR2; Figure S7). HF perfused microtissues showed the most significant transcriptional alteration from Day 1 to Day 5 (Figure 6), with increased expression of a wide group of genes, including pacemaker channels (HCN2 and HCN4; Figure S4), sodium channels (SCN1B; Figure S6), potassium channels (KCND3 and KCNH2; Figure S6), calcium cycling apparatus (CACNA1C, SCL8A1, ATP2A2, RYR2, and CASQ2; Figure S7), sarcomere structure (MYH6, MYH7, MYBPC3, TNNC1, TNNT2, TNNI3, and ACTC1; Figure S3), cell membrane and cytoskeleton (CAV3, JPH2, and ACTB; Figure S8) and metabolism regulators (PPARGC1A; Figure S9).

On Day 5, both perfused microtissues (LF and HF) showed increased B‐type natriuretic peptide (NPPB; Figure S11) and expression compared with static conditions. However, HF perfused microtissues at Day 5 presented with the most notable transcriptional shift when compared with time‐matched static and LF conditions (Figure 6), with higher expression of connexin 43 (GJA1; Figure S10), pacemaker channels (HCN2 and HCN4; Figure S4), potassium channels (KCND3 and KCNH2; Figure S6), calcium cycling (CACNA1C, SCL8A1, ATP2A2, RYR2, and CASQ2; Figure S7), sarcomere structure (MYH6, MYH7, MYBPC3, TNNC1, TNNT2, TNNI3, and ACTC1; Figure S3), cell membrane and cytoskeleton (CAV3 and JPH2; Figure S8), and metabolism (PPARGC1A; Figure S9).

## 4 DISCUSSION

Elucidating the mechanisms involved in tissue morphogenesis and maturation is fundamental for the advancement of tissue engineering. Over time, it has become widely accepted that the foundation of structured, functional tissue not only relies on the biochemical conditioning of the environment but also on the application of relevant physical stimuli (Marsano et al., 2016; Mathur et al., 2015; Ronaldson‐Bouchard et al., 2018; Visone et al., 2018; Vunjak‐Novakovic et al., 2010). When it comes to cardiac tissue engineering, the effects of mechanical and electrical stimuli mimicking the native myocardium have already proved to enhance the in vitro process of heart tissue development (Marsano et al., 2016; Visone et al., 2016, 2018). In vitro models presenting a high density of metabolically active cells of the heart also require a controlled level of nutrients and gases through all the volume of the construct (Radisic et al. 2004, 2006; Richards et al., 2020). Specifically in microfluidic platforms for cardiac tissue engineering, there are already systems that include cell culture medium perfusion (Kobuszewska et al., 2017; Mathur et al., 2015). However, in these studies, aside from the use of less practical off‐chip fluid handling setups, a systematic investigation of the impact of perfusion on modulating the development of cardiac constructs features (i.e., cell structure, construct functionality in terms of contractile behavior, and gene expression) was lacking.

This study describes a heart‐on‐chip platform able to culture and perfuse 3D constructs under controlled flow profiles. We exploited the platform to investigate the influence of flow rate on cardiac microtissues structural and functional maturation. The integration of an on‐chip peristaltic pump represents an attractive approach compared with traditional bulky electro‐mechanical off‐chip fluid handling systems since integrating microfluidic valves to control the flow rate allows to maintain cell culture medium and biological components under the controlled atmosphere of a CO_2_ incubator. In contrast, the electronic components (e.g., solenoid valves, microcontroller, interface) remain outside the humidified environment. Additionally, a constantly pressurized reservoir also acts as a preventive measure in degassing the medium through the gas permeable walls of tubing and PDMS device. This represents a unique feature since air bubbles during perfusion in capillary systems are among the most recurring issues, especially in cell culture, sometimes compromising long‐lasting experiments (Sung & Shuler, 2009). The reservoir capacity can be adapted to the requirements of the system, offering some versatility in terms of medium available through the entire experimental period. Quake's valves‐based microfluidic pumps confer compactness and portability to the entire system while keeping it fully automated, with a precision level comparable to commercial fluid handling systems (Gómez‐Sjöberg et al., 2007; Kellogg et al., 2014; Unger et al., 2000).

The present heart‐on‐chip platform design allows mass transport on the channels flanking a central culture chamber populated with a metabolically demanding construct. This fibrin hydrogel preparation has dense and uniformly distributed cardiomyocytes through the tissue volume and allows cellular and architectural visualization through standard microscopy techniques. Unlike previous microfluidic platforms that also include perfusion of cell culture medium (Kobuszewska et al., 2017; Mathur et al., 2015), we compared statically and dynamically cultured 3D cardiac tissues and investigated how cells handle different flow rates. It was observed that tissues cultured with higher flow rates resulted in enhanced structural and functional maturation. Perfusion had a profound effect on the transcriptional regulation of microtissues. In particular, HF perfusion was associated with the upregulation of a wide group of genes with a critical role in cardiomyocyte physiology, suggesting an improved microtissue maturation. This follows functional data, showing increased microtissue contractile performance at Day 5 with HF perfusion.

Several factors contribute to cardiac tissue maturation and performance in vivo and in vitro (Mills & Hudson, 2019). Perfusion, specifically in HF conditions, was able to increase the expression of key genes in cardiac morphologic and functional maturation, namely: (i) intercellular communications (Connexin 43); (ii) automaticity (HCN channels); (iii) myocyte repolarization (K + channels), (iv) sarcomeric structure (myosin heavy chains, sarcomeric actin, troponins, and regulatory myosin binding protein C), (v) calcium cycling and sensitivity (SERCA2, Calsequestrin‐2, Junctophilin‐2, S100A, RYR2), and (vi) metabolism and mitochondrial biogenesis (PGC‐1A).

Mechanotransduction is the main regulator of natriuretic peptides expression. B‐type natriuretic peptide (BNP) is the main form expressed in ventricular myocytes and sensitive marker of cardiomyocyte stretch (Kerkelä et al., 2015). Therefore, increased BNP expression in HF perfused microtissues at Day 5 suggests that perfusion‐related cardiomyocyte stretch might underlie, at least partially, the observed improved functional performance and maturation.

In addition to mechanical stimulation, we hypothesized that perfusion could also improve oxygen and nutrient delivery. Hypoxia‐inducible factor 1 alpha (HIF‐1α) is a key player in the response to hypoxia (Majmundar et al., 2010), in charge of for the activation of protective transcriptional profile (e.g., VEGF A upregulation). Indeed, microtissue perfusion was able to downregulate HIF‐1a and VEGF, suggesting an improved oxygen delivery to microtissues and protection to hypoxia, which might also contribute for the improved functional performance.

## 5 CONCLUSIONS

On‐chip peristaltic pumping leverages the fluid handling conditions and convenience when applied to cell culture environments while keeping the automated and accurate control of cell culture medium. The microfluidic platform here reported provides the opportunity to fine control different perfusion profiles simultaneously and study their effect on tissue maturation.

The influence of flow rate on cell morphology, cellular organization, tissue responsiveness to pacing, and contractile behavior was investigated together with a comprehensive analysis of relevant gene expression. The employment of such device could be of paramount importance to optimize in a more extensive manner the flow rate conditions in dynamic cultures and consequent generation of more robust in vitro cardiac models.

Moreover, with the vision of increasing the complexity of the described platform, its design was kept compatible for further integrations with other relevant features (i.e., uniaxial cyclic strain and electrical stimulation). This perfused heart‐on‐chip platform thus represents an innovative tool to unravel the role of different environmental cues in cardiac tissue formation.

## CONFLICT OF INTERESTS

The authors declare that there are no conflict of interests.

## AUTHOR CONTRIBUTIONS

Daniela Cruz‐Moreira performed all experimental work, including conception and microfabrication of the microfluidic chip and biological experiments, drew the figures and analysed the data. Roberta Visone assisted in the isolation, culture and pacing of NRVM and data analysis. Francisco Vasques‐Nóvoa performed the experimental work and analysed the data regarding transcriptional profiles. António S. Barros carried out the statistical analysis. Adelino Leite‐Moreira, Alberto Redaelli, and Matteo Moretti contributed in the discussion and interpretation of the results. Marco Rasponi conceived and supervised the project. All authors commented, extended, and improved the manuscript. All authors reviewed and approved the final version of the manuscript.

## Supporting information

Supporting information.

## Data Availability

The data that support the findings of this study are available from the corresponding to other researchers in the field on request.
