# Supplementary material for: Assessing the influence of perfusion on cardiac microtissue maturation: A heart‐on‐chip platform embedding peristaltic pump capabilities
Source: Biotechnol Bioeng. 2021 Jun 1;118(8):3128–37. doi: 10.1002/bit.27836 (PMC8362142; doi:10.1002/bit.27836)
Supplement: Supplementary file 1 — Supporting information. [file BIT-118-3128-s001.pdf]

Supplementary information

Assessing the influence of perfusion on cardiac microtissue maturation: a heart-on-chip platform embedding peristaltic pump capabilities

Daniela Cruz-Moreira,[a†] Roberta Visone,[a] Francisco Vasques-Nóvoa,[b,c,d] António S. Barros,[b,c] Adelino Leite-Moreira,[b,c] Alberto Redaelli ,[a] Matteo Moretti,[e,f] and Marco Rasponi [*a]

Affiliations:

a. Department of Electronics, Information and Bioengineering, Politecnico di Milano, Piazza Leonardo da Vinci 32, Building #21, 20133 Milano, Italy

b. Cardiovascular Research and Development Center, Faculty of Medicine of the University of Porto, Porto, Portugal

c. Department of Surgery and Physiology, Faculty of Medicine, University of Porto, Alameda Prof. Hernani Monteiro, 4200-319 Porto, Portugal

d. Instituto de Investigação e Inovação em Saúde (i3S), University of Porto, R. Alfredo Allen, 4200-135 Porto, Portugal

e. Cell and Tissue Engineering Laboratory, IRCCS Istituto Ortopedico Galeazzi, via Galeazzi, 4, 20161, Milan, Italy.

f. Regenerative Medicine Technologies Lab, Ente Ospedaliero Cantonale (EOC), Via Tesserete 46, 6900, Lugano, Switzerland.

† Current address: 3B's Research Group – Biomaterials, Biodegradables and Biomimetics, University of Minho, AvePark - Parque de Ciências e Tecnologia, Zona Industrial da Gandra, 4805-017 Barco, Portugal

Corresponding author

Marco Rasponi
Piazza Leonardo da Vinci 32
Building #21
20133 Milano, Italy.
Tel.: +39 02 2399 3377;
Fax: +39 02 2399 3360.
Email: marco.rasponi@polimi.it

Supplementary figures

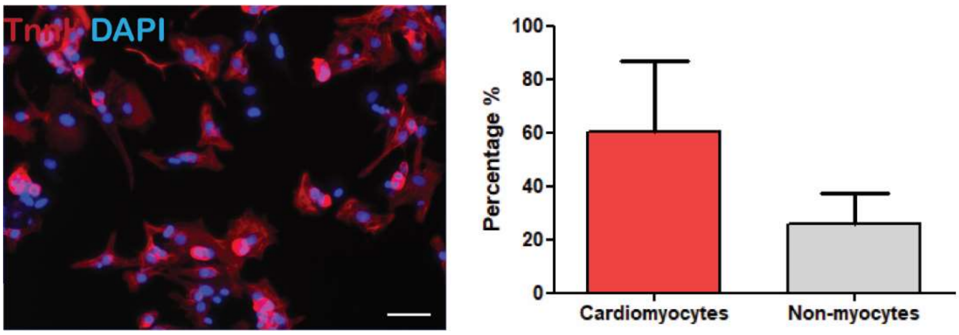

Fig. S1 | Characterization of initial population
Representative fluorescence microphotography of the initial cell population (left), representing cardiomyocytes as positive for troponin I marker. Scale bar: 20µm. On the right, it is represented the percentage of cardiomyocytes assessed by immunostaining before injection within the microchip (n=4).

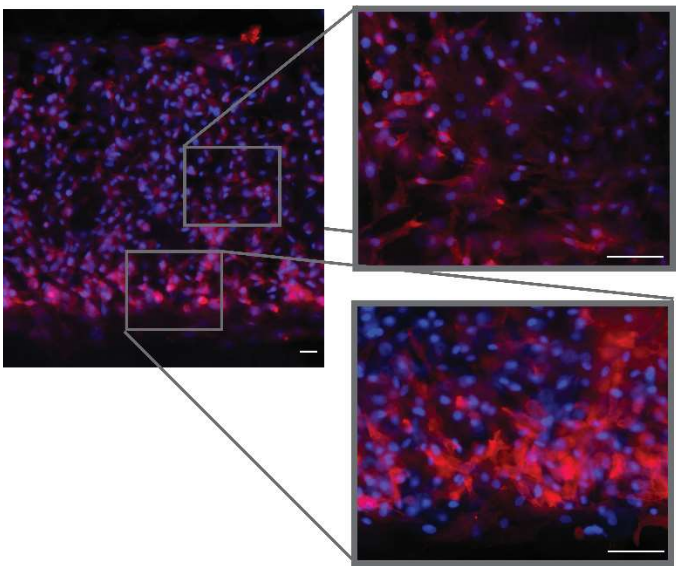

Fig. S2 | Cardiac microtissue
Representative immunofluorescence microphotographs of cardiac microtissues cultured within on-chip pump platform. Cardiac microtissues were stained for Troponin I (red) and DAPI (blue) at day 3. Scale bar:100µm

Sarcomeric Genes

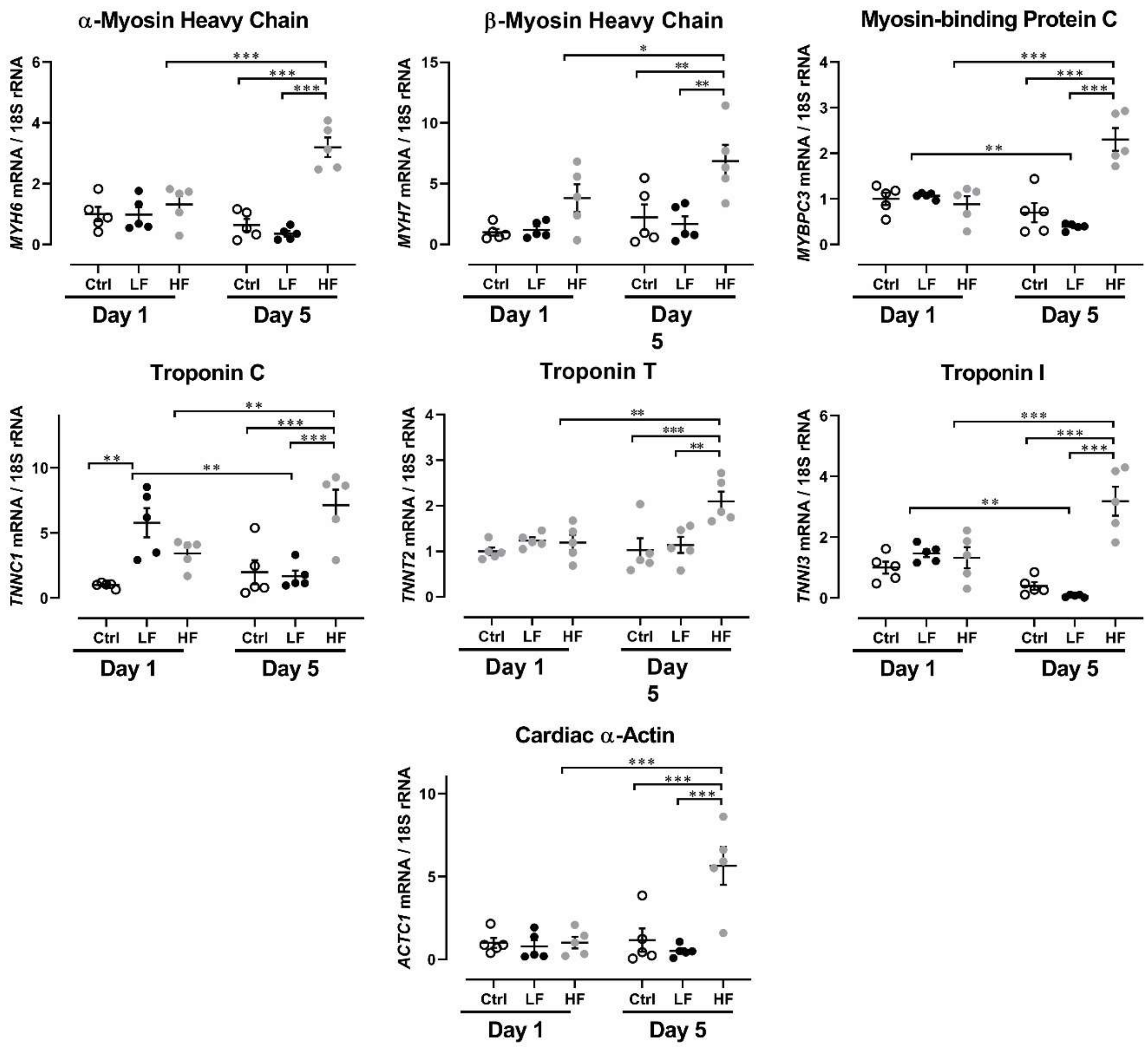

Fig. S3 | Sarcomeric gene expression

***MYH6** – α-Myosin Heavy Chain; **MYH7** – β-Myosin Heavy Chain; **MYBPC3** – Myosin binding protein C 3; **TNNC1** – Troponin C; **TNNT2** – Troponin T; **TNNI3** – Troponin I; **ACTC1** – Cardiac α -actin. N = 5 biologically independent samples / group. Data are represented by mean +/- SEM. *p< 0.05, **p< 0.01, ***p< 0.001*

Pacemaker channels

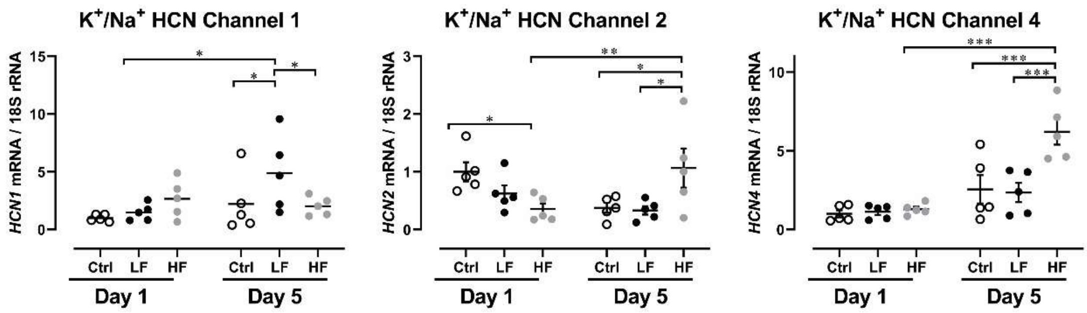

Fig. S4| Hyperpolarization-activated Cyclic Nucleotide–gated (HCN) Channel Gene Expression
HCN1 *– HCN Channel 1;* ***HCN2*** *– HCN Channel 2;* ***HCN4*** *– HCN Channel 4. N = 5 biologically independent samples / group. Data are represented by mean +/- SEM. *p< 0.05, **p< 0.01, ***p< 0.001*

Hypoxia

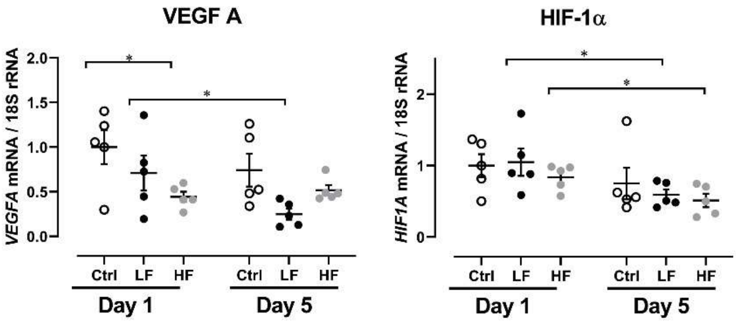

Fig. S5| Hypoxia-driven gene expression

HIF1A *- Hypoxia-inducible factor 1-alpha;* ***VEGFA*** *- Vascular endothelial growth factor A. N = 5 biologically independent samples / group. Data are represented by mean +/- SEM. *p< 0.05, **p< 0.01, ***p< 0.001*

Sodium channels

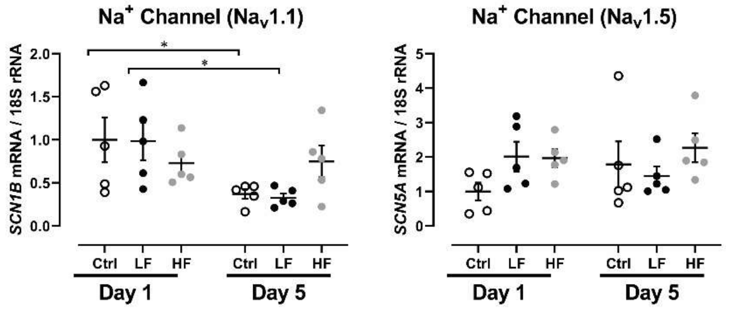

Potassium channels

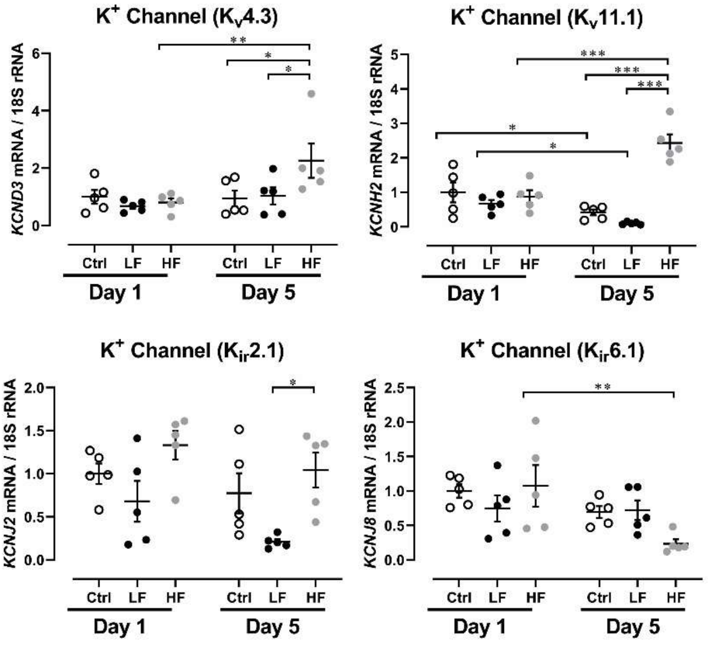

Fig. S6| Sodium and potassium channel gene expression

SCN1B *– Sodium Voltage-Gated Channel Beta Subunit 1;* ***SCN5A*** *- Sodium Voltage-Gated Channel Alpha Subunit 5;* ***KCDN3*** *- Potassium Voltage-Gated Channel Subfamily D Member 3;* ***KCNH2*** *- Potassium Voltage-Gated Channel Subfamily H Member 2;* ***KCNJ2*** *- Potassium Voltage-Gated Channel Subfamily J Member 2;* ***KCNJ8*** *- Potassium Voltage-Gated Channel Subfamily J Member 8. N = 5 biologically independent samples / group. Data are represented by mean +/- SEM. *p< 0.05, **p< 0.01, ***p< 0.001*

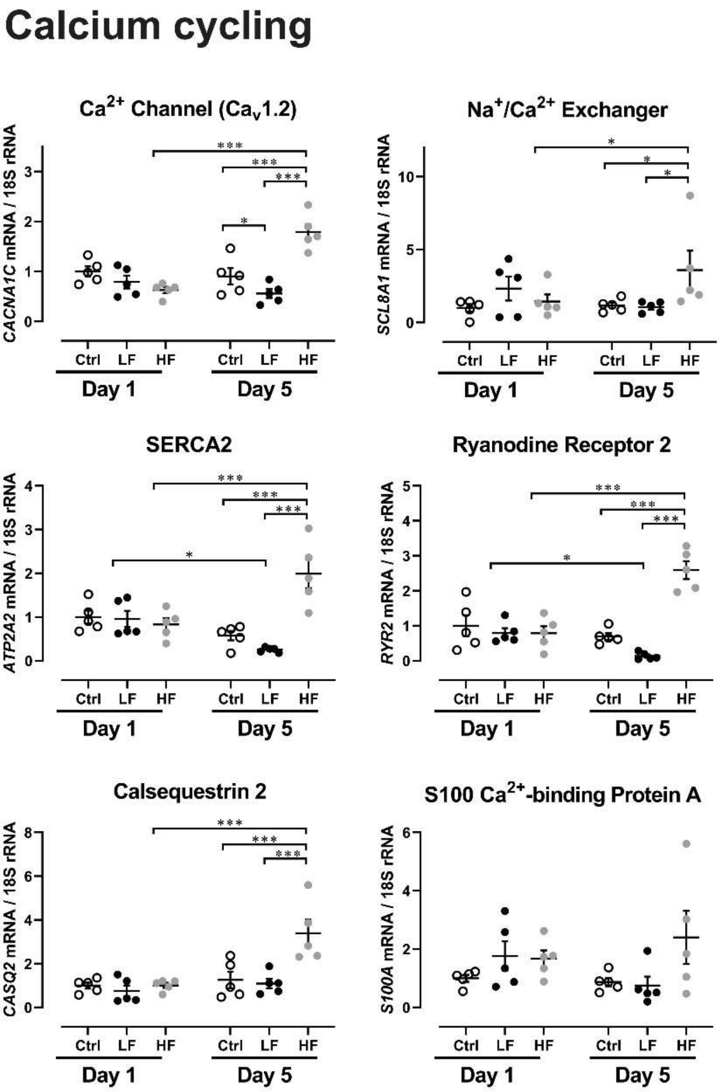

Fig. S7| Calcium cycling apparatus gene expression

CACNA1C *– L-type Calcium Channel;* ***SLC8A1*** *– Sodium-Calcium Exchanger;* ***SERCA2*** *- Sarco/Endoplasmic Reticulum Ca2+-ATPase 2;* ***RYR2*** *– Ryanodine Receptor 2;* ***CASQ2*** *- Calsequestrin 2;* ***S100A*** *– S100 Ca2+-binding Protein A. N = 5 biologically independent samples / group. Data are represented by mean +/- SEM. *p< 0.05, **p< 0.01, ***p< 0.001*

Cell membrane and Cytoskeleton

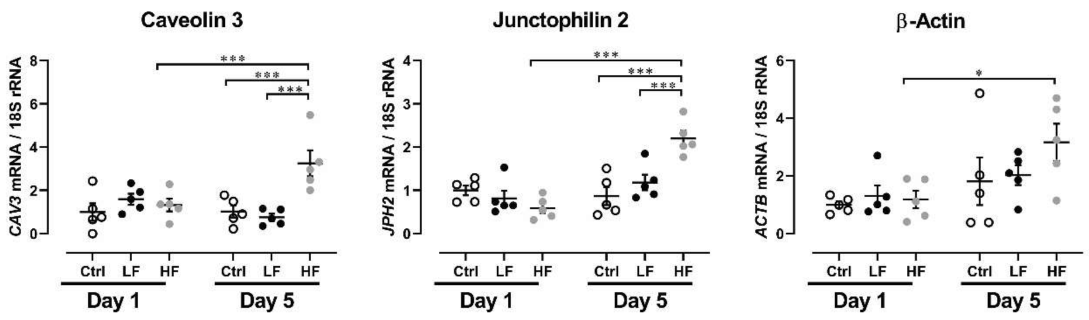

Fig. S8| Cell membrane and cytoskeleton gene expression

CAV3 *– Caveolin 3;* ***JPH2*** *– Junctophilin 2;* ***ACTB*** *– β-Actin. N = 5 biologically independent samples / group. Data are represented by mean +/- SEM. *p< 0.05, **p< 0.01, ***p< 0.001*

Metabolism

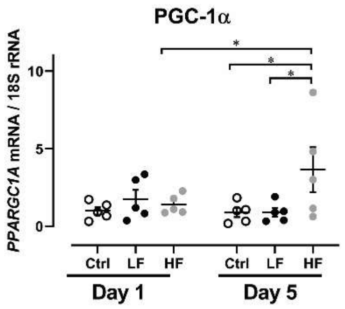

Fig. S9 | Metabolism-related gene expression

***PPARGC1A** - Peroxisome proliferator-activated receptor gamma coactivator 1-alpha. N = 5 biologically independent samples / group. Data are represented by mean +/- SEM. *p< 0.05, **p< 0.01, ***p< 0.001*

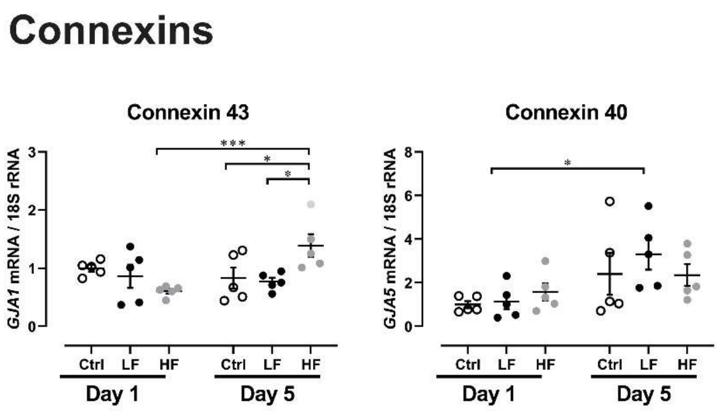

Fig. S10| Connexin gene expression

*GJA1 – Connexin 40; GJA5 – Connexin 43. N = 5 biologically independent samples / group. Data are represented by mean +/- SEM. *p< 0.05, **p< 0.01, ***p< 0.001*

Natriuretic peptides

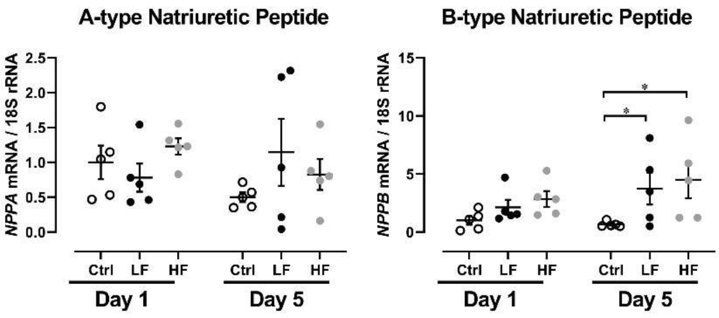

Fig. S11| Natriuretic peptide gene expression

*NPPA – A-type Natriuretic Peptide; NPPB – B-type Natriuretic Peptide. N = 5 biologically independent samples / group. Data are represented by mean +/- SEM. *p< 0.05, **p< 0.01, ***p< 0.001*

Supplementary Table S1

Gene	Forward Primer 5' → 3'	Reverse Primer 5' → 3'
18S	CGT CTG CCC TAT CAA CTT TCG	CTT GGA TGT GGT AGC CGT TT
ACTB	ATG GTG GGT ATG GGT CAG A	CTC ATT GTA GAA AGT GTG GTG CC
ACTC1	GCC AAC CGT GAG AAG ATG ACA	GCC AGA ATC CAG AAC AAT GCC
ATP2A2	CAC TGC GCT GGG GTT CAA TCC TC	GCA GCG CCA ACA TAA CAG CCA ATA
CACNA1C	CCC ATG CCG GAA GCC AGT GC	TCA GCC GCC AGG GAG AT
CASQ2	TCA TCC CTA ACA AGC CCT ACA CAG	TCT TCT TCC GCA AAT GCC ACA ATG
CAV3	GTG AAC AGA GAC CCC AAG AAC	ACG CCA TCG AAG CTG TAA GTG
GJA1	CGC GCC GGC TTC ACT TT	CCC CAG GAG CAG GAT TCT
GJA5	AGG GCG GAG GAA AGG AAG CAG AA	GCG GAA AAT GAA CAG GAC GGT GAG
HCN1	GGA GCT GTG GGG AAG AAA ATG TAT	AGT GCG CCG GCC CTT GGT CAG C
HCN2	CAG GGG AAG ATG TTT GAC GAG GAC	CAG CAT GGC GGT GAC GAA GTT
HCN4	GGG CGC CAC CTG CTA TGC	CGT GGA TGC GCT GCC TGG TGT
HIF1A	CAA CTG CCA CCA CTG ATG	CCA CTG TAT GCT GAT GCC TTA
ITPR3	CCC CGC CTT CGA TTC CTC TAC TGC	ACC CCC TTC GTC ATC CAC CTC TA
JPH2	GCC GAG GCC GAG GTG GAG GTA GA	AGC CAG GCC GAT GTT CAG CAG GAT
KCND3	CCG CCT TGC CAG AAT CCG TGT G	GGT CTT GCC CAT GTG CTC CTC TTC
KCNH2	CAC GTC GCG CCG CAG AAC ACC TT	GCC CGA GAG TAG CCG CAC AGT
KCNJ2	CAC GGC TGC CTT CCT CTT CTC C	GAT GAT GCA GCC CAC GAT TGA CT
KCNJ8	GCC CAG GCC CAC AGA AG	GCA CCG AGG CGC TAA TGA T
MYBPC3	CCC CTG ACG ACC CTA TTG	TGA GAA GAC GAT GCT GCC G
MYH6	GCT GGG CTC CCT GGA CAT TGA CC	CAG CCC CGC CTT GAA GAA CAC CT
MYH7	CTT CCC CAA CCG CAT TCT TTA T	CTT CTC AGC CCC TTT CCG
NPPA	GGG CTC CTT CTC CAT CAC CA	ATC TTC TAC CGG CAT CTT CTC CTC
NPPB	GGA TTG GCG CAG TCA GTC GTT	CCG CAG GCA GAG TCA GAA G
PPARGC1A	CAG CCA CTC CAC CAA GAA AG	TCA CCA AAC AGC CGT AGA CT
RYR2	GGA GCG TGG CCC CTA TCA GC	AGC CGG AGA ACA TCA CCA CCA AT
S100A	AGA CCC TCA TCA ATG TGT TCC	GGA GAG TTC AGT TTG TAG CAG G
SCN1B	GTG GTG GGC GCG GTG CTG GTA TC	CGT GAA GGT CTC GGC GGT GGT CTC
SCN5A	ACC GAC CCC CAG ACA CGA CCA CT	TCC GGG CTC CTC AAA ACC ATC TG
SLC8A1	AGA CCC AGA AGG AAA TCA GAG T	AGA CAA GCA ATC GCA GAC ACG
TNNC1	CAG AAC CCC ACA CCT GAG	ACT TCC CTT TGC TGT CAT CCT
TNNI3	CAG CCC TTG GTG TTG GAT G	CAG TGA TGT TCT TGG TGA CTT TGC
TNNT2	GAG GCA GTG GAG GAG GAG GAT GG	TGC TGG GCT TGG GTT TGG AGT C
VEGFA	CAA CTT CTG GGC TCT TCT CTC	CTT CTC CTC TTC CTT CTC TTT C

Supplementary methods

Photolithography

Two 4'' silicon wafers (100mm diameter, 0-100Ω-cm, 500µm thickness, test grade, University wafer) were treated with a thin film of Ti-Prime adhesion promoter (MicroChemicals GmbH). Cell culture chamber was produced by a two-step process with different photoresists: (i) AZ 40XT positive photoresist (Merck KGaA) was used to create round valve features and (ii) SU8-2050 (MicroChemicals GmbH) for the cell culture chamber. To obtain the round valves features, AZ 40XT was spin-coated and post baked according to suppliers' instructions.

Subsequently, the high-resolution transparency mask containing the valves design was aligned on the wafer using Karl Süss Mask Aligner MA6/BA8 (Süss MicroTec) and the photoresist was exposed to 414 mJ/cm^2 of collimated UV light. The photoresist underwent post-exposure bake and was developed in AZ 726 MIF (Merck KGaA). Finally, the valve structures were reflowed and hard-baked at 190ºC overnight. Subsequently, SU8 2035 negative photoresist was spin-coated 100µm high channels and chambers and then soft baked. The high-resolution transparency mask containing the chamber design was aligned on the wafer and the negative photoresist was exposed to 80 mJ/cm^2 of collimated UV light. The photoresist underwent post-exposure bake and was developed in SU8 Developer (MicroChemicals GmbH).

For the manufacturing of the control master mould, a second wafer was coated with 20-25µm thick layer of SU8 2010 and soft-baked. A high-resolution mask containing the control layer features was aligned on top of the wafer and the photoresist was exposed to 184 mJ/cm2 of collimated UV light, following post-exposure bake and development. Finally, it was hard-baked for 2 minutes at 120°C.

Multilayer soft lithography

PDMS-based device was produced through multilayer soft lithography. A two-part PDMS kit (Sylgard 184, Dow Corning) was used to create the liquid polymer. The cell culture chamber layer was made by pouring uncured liquid PDMS (5:1 elastomer:crosslinker ratio) on the chambers mould to a thickness of 5mm. The control layer was made by spin-coating uncured PDMS (20:1) elastomer: crosslinker ratio onto the control mould to form a 35-40 µm thick layer. Both layers were pre-cured for 20 minutes at 80°C. The thick cell culture chamber layer was peeled off its mould, access points were created for inlet, outlet and ports for hydrogel injection. The thick layer was aligned on top of the thin layer on the wafer and baked at 80ºC overnight, to achieve irreversible bonding. Subsequently, the layers were peeled off, access ports for the control layer were created and assembled by air plasma bonding (Harrick Plasma Inc). The ports to access the inlet and control channels permit the connection of 23-gauge stainless steel couplers (Instech Laboratories), which fit tightly into both the ports on the chip and the inside of the tubing, for both the chamber and control layers. The final assembled device was further cured at 80°C for 15minutes to finalize the bonding process.

Evaluation of flow output

Briefly, dead-ended channels that constitute the control layer were filled entirely with ddH_2O. The cell culture channels were primed with ddH_2O until the fluid front inside the tygon tube was visible. Complete wetting of the fluidic channel reduced the potential for bubble formation during pump operations. The pump controller software ran for a controlled period of time and the displacement of the fluid front in the tube was measured. Volumetric flow rate was calculated as a cylindrical volume as a function of the distance travelled by the fluid front in one minute:

$$Q = \frac{\pi \times d^2 \times \Delta x}{4t}$$

Where Q represents the flow rate, d the tubing inner diameter, Δx the displacement of the fluid front, and t the pumping time.

The driving frequency of a complete cycle was set as 0.2 Hz (1000 ms of delay in-between cycle steps), 0.4 Hz (500 ms delay), 0.67 Hz (300 ms delay), 1 Hz (200 ms delay), 2 Hz (100 ms delay), 4 Hz (50 ms delay). Even though the solenoid valves had a theoretic limitation of 4ms in time response, the minimum value of actuation delay, chosen for the experiment, was equal to 50 ms. For statistical purposes, three tests were performed at each frequency and, for each test, three measurements were evaluated and analysed. These values were analysed using scientific graphing and statistic software Prism (GraphPad Software Inc.) and a curve that relates flow rate and pumping cycle frequency was created using the same software.
